# The Dynamics of the Bacterial Community of the Photobioreactor-Cultivated Green Microalga *Haematococcus lacustris* during Stress-Induced Astaxanthin Accumulation

**DOI:** 10.3390/biology10020115

**Published:** 2021-02-04

**Authors:** Konstantin Chekanov, Anna Zaytseva, Ilgar Mamedov, Alexei Solovchenko, Elena Lobakova

**Affiliations:** 1 Faculty of Biology, Lomonosov Moscow State University, 1-12 Leninskie Gory, 119192 Moscow, Russia; kublanovskaya@mail.bio.msu.ru (A.Z.); solovchenko@mail.bio.msu.ru (A.S.); elena.lobakova@gmail.com (E.L.); 2 Centre for Humanities Research and Technology, National Research Nuclear University MEPhI, 31 Kashirskoye Highway, 115522 Moscow, Russia; 3 Shemyakin and Ovchinnikov Institute of Bioorganic Chemistry, Russian Academy of Sciences, 117997 Moscow, Russia; imamedov78@gmail.com; 4 Institute of Natural Sciences, Derzahvin Tambov State University, 39200 Tambov, Russia

**Keywords:** *Haematococcus lacustris*, astaxanthin, microbial communities, photobioreactors

## Abstract

**Simple Summary:**

The microalga *Haematococcus lacustris* is a source of the natural colorant astaxanthin, a powerful antioxidant and key component of cosmetics and animal feed. *Haematococcus* is cultivated in photobioreactors. It can obtain energy just from a light illuminating photobioreactor and uses inorganic salts and CO_2_ as sources for chemical elements. The most widespread approach for *Haematococcus* cultivation is the two stage scheme. At the first stage, biomass accumulation under favorable growth conditions occurs. At the second stage, the cells are subjected to stress inducing astaxanthin synthesis. Generally, the culture of *Haematococcus* is not axenic. It exists in the form of a community with bacteria constituting its microbiome. The information on photobioreactor-cultivated *Haematococcus* microbiome is scarce. We analyzed its dynamic during astaxanthin production by DNA metabarcoding and microscopic observations. The main results of the work include the characterization of the daily dynamic of this microbiome and the revealing of contact between microalgae and bacteria. These findings are of potential significance for biotechnology. On one hand, they provide an insight into possible bacterial contamination of the harvested algal biomass. On the other hand, they reveal the presence of a core microbiome or bacteria essential for the growth of the microalga existing in all *Haematococcus* cultures.

**Abstract:**

*Haematococcus lacustris* is a natural source of a valuable ketocarotenoid astaxanthin. Under autotrophic growth conditions, it exists in the form of a community with bacteria. The close coexistence of these microorganisms raises two questions: how broad their diversity is and how they interact with the microalga. Despite the importance these issues, little is known about microorganisms existing in *Haematococcus* cultures. For the first time, we characterize the dynamic of the *H. lacustris* microbiome of the microbiome of *Haematococcus* (a changeover of the bacterial associated species as function of the time) cultivated autotrophically in a photobioreactor based on 16S rRNA metabarcoding data. We found that Proteobacteria and Bacteroidetes are predominant phyla in the community. The Caulobacter bacterium became abundant during astaxanthin accumulation. These data were supported by microscopy. We discuss possible roles and interactions of the community members. These findings are of potential significance for biotechnology. They provide an insight into possible bacterial contamination in algal biomass and reveal the presence of bacteria essential for the algal growth.

## 1. Introduction

*Haematococcus lacustris* (Volvocales, Chlorophyceae) is a unicellular green alga; a biotechnological source of the ketocarotenoid astaxanthin and a natural pigment sought after in the market for use in cosmetics, functional food and animal feed [1,2,3,4,5,6,7]. To produce astaxanthin, *H. lacustris* is cultivated at an industrial scale because it accumulates the pigment in up to 4–5% of dry cell weight [3,4,5,6,8]. In different reports, the conditions of *Haematococcus* cultivation (temperature, light intensity and spectral composition, media composition and the bioreactor design) vary widely [8,9,10,11,12]. *H. lacustris* can grow under autotrophic, mixotrophic and heterotrophic conditions [1,2,9,10,11,12,13] using acetate as an organic carbon source [2,9]. The growth on malonate [2] and glucose [14] as a single carbon source has been also reported. Photosynthesis plays a key role in the energy and photofixed carbon supply in the cells of *H. lacustris* under mixotrophic and autotrophic conditions [2,15,16]. As a rule, astaxanthin biosynthesis is induced by diverse stressors such as nitrogen and/or phosphorus starvation, bright light, high salinity and reactive oxygen species [1,4,6,9,15]. The stressed *H. lacustris* cells are transformed to metabolically inactive aplanospores referred to as haematocysts [4,17]. *H. lacustris* also accumulates the pigment in response to treatment with phytohormones promoting the metabolic quiescence, e.g., abscisic acid [18,19] and C_2_H_4_ [20,21]. The most widespread approach for astaxanthin production using *Haematococcus* is the biphasic (or two stage) cultivation [8,22]. At the first “vegetative” stage the conditions are favorable for cell division and for biomass accumulation. At the second stage, the “inductive phase”, the vegetative cells are subjected to stressful conditions inducing astaxanthin accumulation.The heterotrophic and mixotrophic cultivation of *H. lacustris* are carried out aseptically. Large-scale cultures of *H. lacustris* often suffer from contamination by other microalgae, heterotrophic bacteria and eukaryotes [3,5,7,11]. Even under aseptic conditions, it is very difficult to maintain axenic *H. lacustris* cultures [11]. As a rule, *H. lacustris* exists in association with other microorganisms, especially bacteria. The close coexistence of the microalga and other microorganisms raises two questions: (i) how broad their diversity is and (ii) how they interact with the microalga.Despite the importance of this issue, little is known about the microorganisms existing in *H. lacustris* monoalgal cultures except for a few reports on the parasitic micromycetes damaging industrial algal cultures [23]; the blastoclad *Paraphysoderma* specifically interacts with *H. lacustris* cells attacking it on a certain stage of its lifecycle [24]. Previously, we described natural microbial communities formed around *H. lacustris* from the White Sea coastal rock baths [25,26,27]. As with *H. lacustris* itself, many microorganisms from these communities are characterized by their resilience to adverse environmental conditions. Filamentous cyanobacteria are particularly known for their stress tolerance and are frequently abundant in these habitats [25]. Cyanobacteria and *H. lacustris* are dominant photosynthetic organisms in these biotopes [25,26]. Natural algal communities might also include grazing protists, e.g., *Vermamoeba*, *Paravahlkampfia*, ciliates [27]. The prokaryotes common for *H. lacustris*-based natural communities from the White Sea are dominated by *Comamonadaceae* and also include representatives of the families *Cytophagaceae*, *Xanthomonadaceae*, *Acetobacteraceae*, *Rhodobacteraceae* and *Rhodocyclaceae* [26]. Isolation under laboratory conditions and the removal of the cyanobacterial and eukaryotic consorts changes the bacterial diversity of *H. lacustris* cultures and shifts the dominant taxa.

In this work, we dissected the composition of the bacteriome of *H. lacustris* laboratory cultures and followed its changes as a function of cultivation conditions and growth stage. Special attention was paid to the effect of the induction of astaxanthin biosynthesis by the stress and recovery of vegetative growth on the composition of the bacterial community of *H. lacustris* cultures autotrophically grown in a photobioreactor. The investigation of bacteria from H. lacustris cultures provides valuable data about the satellites of this microalga. These data may be used for the construction of stable associations of H. lacustris and bacteria. This innovation can reduce the contamination of *H. lacustris* cultures during laboratory and industrial cultivation and increase the stability of the microalgal culture.

## 2. Materials and Methods

### 2.1. Algal Strain, Cultivation Conditions and Sampling

The strain *H. lacustris* BM1 (IPPAS H-2018) isolated from the White Sea coastal zone [28] was used. The culture was maintained in glass column photobioreactors in 400 mL of a mineral BG-11 medium [29] bubbled by an air-gas mixture containing 5% (*v*/*v*) CO_2_ under the conditions conducive for the vegetative growth [30] in the laboratory.

Vegetative cells sampled at the exponential growth phase were subjected to stress for the induction of astaxanthin biosynthesis as described previously [30] by increasing irradiance (from 60 to 480 μmol/m^2^/s as measured by a LI-185 cosine-corrected light sensor, LI-COR, USA) and the transferring of the *H. lacustris* cells to a BG-11 medium [31].

The samples for metagenomic analysis and microscopy were taken from the vegetative culture incubated under the conditions conducive for vegetative growth (the sample ‘0 day’). Upon transferring the culture to the stressful conditions, samples were taken daily for five days. The culture was later returned to the initial conditions (conductive for vegetative growth) to study the features of the bacterial community typical of the *H. lacustris* at the recovery phase. At this stage, samples were collected daily for three days. All cell suspension samples (2 mL) were taken and handled aseptically. The samples from the two reactors were randomly pooled together in an equal proportion (1 mL + 1 mL) for further environmental DNA (eDNA) extraction and sequencing. As was shown previously, the pooling of microbiome samples before DNA amplification and metagenomics sequencing in order to estimate community level diversity is a viable measure in population level association research studies [32]. The samples were stored for 1–3 days at −80 °C, which is generally recognized as ideal storage conditions [33] before eDNA extraction.

Three laboratory cultures (LC-I, LC-II, LC-III) of previously identified *H. lacustris* strains BMP/16, BMK/16 and BMM1/16 [34] were taken as a control for the principal component analysis (PCA,) (Table 1). A detailed description of their bacterial composition has been given previously [26]. They were maintained in the cell culture T-75 TC-treated cell culture flasks (Eppendorf, Hamburg, Germany) in 40 mL of a BG-11 medium in the laboratory. The temperature in the laboratories was maintained in the range of 19–25 °C, relative humidity was in the range of 60–80% and laboratory cultures were illuminated by a cold white light (60 μmol/m^2^/s). They were cultured without reseeding for 3–6 months for the identification of stable bacterial components of the microalgae. The samples (2 mL, one replicate) were taken from each flask aseptically.

In addition, an environmental sample MS1-18 [26] of natural *H. lacustris* colonies was used as an outgroup for the analysis. This sample was collected from a supralittoral rock bath in the Probkina Gubka Bay of the Kandalaksha Bay of the White Sea (66°32′24″ N; 33°11′2″ E) from the sample location MS1 in 2018.

To compare bacterial compositions in the *H. lacustris* cultures and in the laboratory, two open 1.5 mL plastic tubes filled with the BG-11 medium were stored in the center of the laboratory over two days. They were then subjected to the same DNA metabarcoding analysis procedure as was used for other samples.

### 2.2. Dry Cell Mass and Pigment Assay

The microalgal sample dry mass was determined gravimetrically [35]. The astaxanthin content of the *H. lacustris* biomass was determined spectrophotometrically in dimethyl sulfoxide extracts [36] according to [37] using an Agilent Cary 300 spectrophotometer (Agilent, Lexington, KY, USA) in the standard 1 cm quartz cuvettes.

###  2.3. Microscopy

#### 2.3.1. Light Microscopy

*H. lacustris* cultures were monitored by bright field light microscopy on a Leica DM 2500 microscope (Leica, Wetzlar, Germany) equipped with a Leica DFC 7000T camera of the same manufacturer. 

#### 2.3.2. Electron Microscopy

*H. lacustris* cultures were studied with scanning (SEM) and transmission (TEM) electron microscopy using a fixation method developed previously for *H. lacustris* [28,38]. Cells were fixed with 2.5% (*v*/*v*) glutaraldehyde and 2% OsO_4_ (*wt*/*v*) in a 0.1M cacodylate buffer (pH 7.4). The samples were then dehydrated in a graded series of ethanol solutions including 100% ethanol. Additionally, the cells were contrasted by 2% (CH_3_COO)_2_UO_2_ in 100% ethanol.

For TEM, the samples were incubated at 56 °C in a series of mixtures of epoxy resin Araldite M (Fluka, Germany), dodecenyl succinic anhydride (DDSA) of the same manufacturer as the hardener and 100% ethanol in following proportions (by volume): Araldite M:DDSA:ethanol (1:1:2), Araldite M:DDSA:ethanol (1:1:0) then for three days at 56 °C in a mixture of 2.7 mL Araldite M and 2.3 mL of DDSA in the presence of a catalyst (2,4,6-*tris*-[(dimethylamino)methyl] phenol) (Fluka, Germany) for the resin polymerization. Ultrathin sections were prepared using an ultramicrotome LKB 4800 (Bromma, Sweden), transferred to palladium nets and additionally contrasted by lead citrate [39]. The cross-sections were studied under a Hitachi HU-11F (Hitachi Ltd., Tokyo, Japan) microscope at an accelerating voltage of 80 kV.

For SEM, the samples were fixed in glutaraldehyde and OsO_4_ according to [28], dried at a CO_2_ critical point in an HCP-2 dryer (Hitachi, Japan), coated by Pd in an IB Ion Coater (Eiko, Japan) and evaluated on a JSM-6380LA (JEOL, Japan) microscope at an accelerating voltage of 15 kV. 

### 2.4. eDNA Isolation and Preparation of 16SrRNA Libraries

eDNA was isolated from the samples using a MagJET Plant Genomic DNA Kit (Thermo Scientific, USA, catalog number K2761) in accordance with the manufacturer’s protocol. eDNA concentration and purity were estimated spectrophotometrically by a NanoDrop 2000C (Thermo Fisher Scientific, Waltham, MA, USA). Amplicon libraries of the V4 fragment of the *16SrRNA* gene were prepared as described previously [25] and sequenced on a MiSeq benchtop sequencer (Illumina, San Diego, CA, USA) using a MiSeq 500 cycles kit (Illumina, USA) for 2 × 250 bp paired-ends sequencing. 

### 2.5. Metagenomic Data Analysis

Primary next generation sequencing (NGS) data were pre-treated in the Trimmomatic tool [40]. Illumina-specific adapter sequences were cut from the reads; reads shorter than 200 bases, longer than 1000 bases and those with an average Phred quality score of lower than 30 in every 30 bases were eliminated from the datasets. Pair-end reads were merged. Chimeric sequences were detected and eliminated by the Chimera.slayer tool [41] in QIIME v. 1.9.1 [42]. The reads quality was checked by FastQC. The subsequent analysis and visualization were conducted using VAMPS (Visualization and Analysis of Microbial Population Structures) software (version 2, https://vamps2.mbl.edu/) [43]. Sequences were packed into operational taxonomic units (OTU) by the single linkage preclustering method [44] using clustering thresholds of 3%. The taxonomic assignment of OTUs was conducted using the Silva 119 database [45]. The sequences with uncertain taxonomy and the sequences corresponding to the chloroplast DNA of Chlorophyta and Streptophyta were excluded from the subsequent analysis.

In order to describe bacterial abundance of the microbiome of *Haematococcus* microalgae, we analyzed α-diversity (diversity of taxa within the sample) and β-diversity (diversity between the samples) across our dataset. To describe the α-diversity, several numeric indices were calculated for each sample. The Shannon entropy index (*H*) [46], widely used for diversity analyzing, was calculated as

$$H=\overset{N}{\underset{i=1}{\sum }}\left(-p_{i}\ln\left(p_{i}\right)\right)$$

where $p_{i}$ was the ratio of the reads corresponding to a taxon *i* to the total read number observed in the sample. The reverse Simpson index (*d*) [47] was calculated as

$$d=1-\overset{N}{\underset{i=1}{\sum }}p_{i}{}^{2}$$

The non-parametric Chao-1 estimator ($N_{Chao-1}$) and abundance-based coverage estimator (*ACE*, $N_{ACE}$) were used to predict the possible number of taxa in the sample. The Chao-1 index [48] based on the correction of the observed taxa number for the taxa that escaped detection assuming the Poisson distribution was calculated as

$$N_{Chao-1}=N+\frac{{\left(n_{1}\right)}^{2}}{2n_{2}}$$

where N was an observed taxa number in the sample and $n_{1}$ and $n_{2}$ were the number of taxa observed 1 and 2 times in the sample, respectively. The ACE estimator [49] based on the number of ‘rare’ taxa $n_{rare}$ (the number of taxa observed ≤ 10 times in the sample) and ‘abundant’ taxa $n_{abund}$ (>10 times in the sample) was calculated as

$$N_{ACE}=n_{abund}+\frac{n_{rare}}{C}+\frac{n_{1}}{C}\gamma^{2},$$

where the coverage estimation (C) and the square of the estimated coefficient of the variation of the relative abundances of the OTUs ($\gamma^{2}$) were calculated based on the number of ‘rare’ taxa ($n_{i}$, observed by i≤10 −times)

$$C=\frac{\overset{10}{\underset{i=2}{\sum }}\left(i\times n_{i}\right)}{\overset{10}{\underset{i=1}{\sum }}\left(i\times n_{i}\right)}.\;\gamma^{2}=\max\left[\frac{n_{rare}}{C}\times \frac{\overset{10}{\underset{i=1}{\sum }}\left(i\left(i-1\right)\times n_{i}\right)}{\overset{10}{\underset{i=1}{\sum }}\left(i\times n_{i}\right)\left(\overset{10}{\underset{i=1}{\sum }}\left(i\times n_{i}\right)-1\right)};0\right]$$

For the β-diversity estimation, the Morisita–Horn dissimilarity index [50] was calculated for each sample pair of *x* and *y*:

$$\chi_{ij}=1-\frac{2\sum_{i}\left(p_{i}^{x}p_{i}^{y}\right)}{\sum_{i}{\left(p_{i}^{x}\right)}^{2}+\sum_{i}{\left(p_{i}^{y}\right)}^{2}},$$

where $p_{i}^{x}$ and $p_{i}^{y}$ were the ratio of the reads corresponding to a taxon *i* to the total read number observed in the samples *x* and *y*, respectively.

A PCA with three components was also applied to the samples. Three control *H. lacustris* cultures isolated from natural samples also from the same region and maintained in the laboratory as well as the environmental *H. lacustris* sample from the White Sea region (see Section 2.1) were taken for PCA as an “outgroup”. 

## 3. Results and Discussion

### 3.1. Astaxanthin Accumulation by the Microalgal Cells

**Figure 1:**
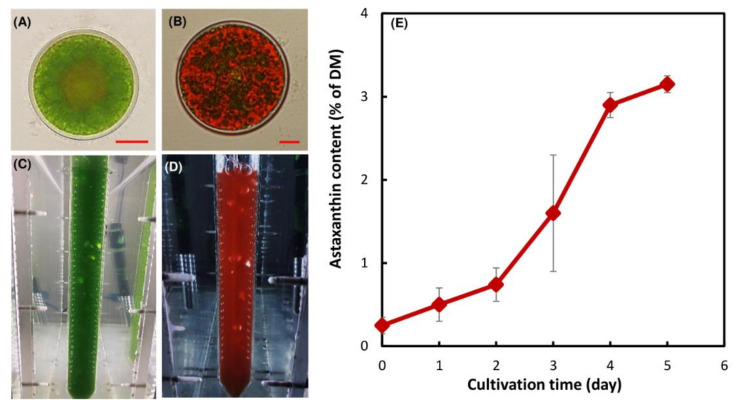
Cells of *H. lacustris* at the vegetative growth phase (**A**) and at day five of the inductive phase (**B**). Cell suspensions of *H. lacustris* cultivated in a photobioreactor under autotrophic growth conditions at the vegetative growth phase (**C**) and at day five of the inductive phase (**D**). Carotenoid accumulation in the *H. lacustris* cells under the stressful conditions; average ± SD (*n* = 3) are shown, DM: dry cell mass (**E**).

On the first day of stress exposure the culture was dominated by the green immotile to carotenoid accumulation. The carotenoid content of the algal biomass at day five of cultivation increased from 0.20 ± 0.05% to 3.2 ± 0.1% of dry cell mass (Figure 1E), which is typical for autotrophically grown *H. lacustris* [1,9,28].

### 3.2. Microscopic Observations of the Bacterial Community

**Figure 2:**
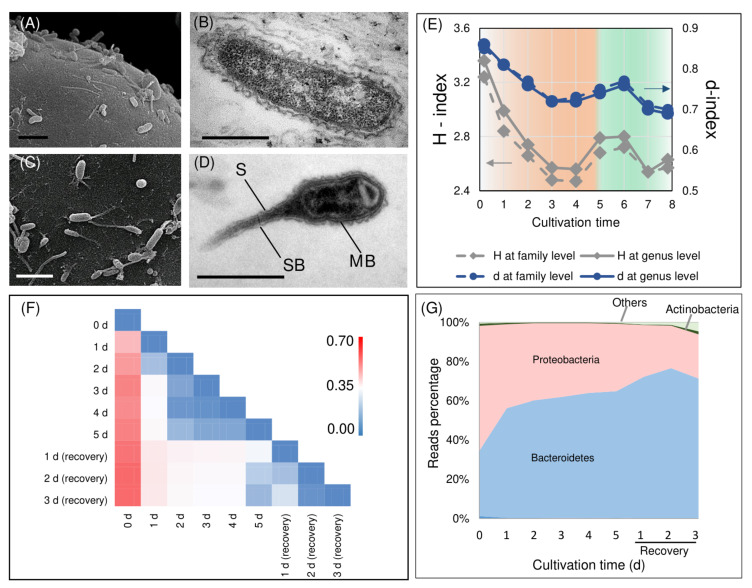
Attachment of bacteria to the surface of *H. lacustris* vegetative cells (**A**) and haematocysts (**C**). Typical rod-shaped gram-negative bacteria abundant in the suspension of *H. lacustris* cells cultivated in a photobioreactor under vegetative growth-promoting conditions (**B**). The *Caulobacter*-like bacteria abundant in the *H. lacustris* cell suspensions cultivated in the photobioreactor under stressful conditions (**D**); S: stalk, SB: stalk band, MB: main body of the cell. The dynamics of Shannon (grey lines, diamonds) and reverse Simpson (blue lines, circles) diversity indexes at genus (solid lines) or family (dashed lines) levels during astaxanthin accumulation and vegetative growth recovery of *H. lacustris* cultivated in a photobioreactor (**E**). The heatmap of the Morisita–Horn index values at the genus level reflects the similarity of the *16SrRNA* gene libraries obtained for the *H. lacustris* bacterial association at different stages of the experiment (**F**). The dynamics of the bacterial consortium formed around the *H. lacustris* bacterial association at different stages of the experiment at the level of phylum (**G**).

Microalgal cells dwelling in an aqueous environment are believed to alter the conditions in the proximity of their cell surface in comparison with the bulk medium. This space is characterized by a peculiar concentration and hydrodynamic force gradients and is referred to as a phycosphere [51], a zone suitable for colonization by bacteria. Most of the bacteria visually discovered in the *H. lacustris* monoalgal cultures cultivated autotrophically in photobioreactors were rod-shaped with a typical structure of gram-negative cells, 0.2–0.6 μm wide and 1–5 μm long (Figure 2B). They were surrounded by two membranes separated by periplasmic space. Abundant ribosomes in their cytoplasm reflected their potentially high metabolic activity. Our microscopy observations showed that rod-shaped bacteria abundant in the *H. lacustris* cultures attached to the surface of *H. lacustris* vegetative cells by their apexes or lateral surface (Figure 2A). A similar phenomenon was observed in *H. lacustris* natural communities [26]. Obviously, the contact between the microalgal and bacterial cells was retained after isolation of the *Haematococcus* culture and its maintenance under laboratory conditions was likely indicative of the interaction between the microalga and bacteria in the culture.

Under the stressful conditions promoting carotenogenesis, a high number of prosthecate bacteria were observed on the surface of *H. lacustris* cells (Figure 2C). They had a 1–3 μm long appendix (prostheca) attached to the *H. lacustris* cells by its distal end. It was surrounded by the cytoplasmic and outer cell membrane similar to the main body of the cell. The prostheca also contained a cytoplasm with ribosomes; in a few cases, pronounced stalk bands were observed (Figure 2C). Prosthecate cells were approximately 0.2–0.3 μm wide and 2–3 μm long (including the stalk), which is in accordance with the description of prosthecate *Caulobacter*-like bacteria [52].

The prosthecate bacteria are abundant in natural oligotrophic waters due to their tolerance to prolonged nutrient shortage, e.g., during incubation in distilled water or under the conditions of N_2_ fixing aerobe enrichment cultures [52,53]. Often *Caulobacter*-like bacteria are attached to the surface of microalgae such as diatoms, chlorophytes, cryptomonads and cyanobacteria [52].

Dimorphic prosthecate bacteria exist in two forms: motile cells with a single flagellum and pili and a non-motile ‘stalked’ form [53,54]. The genes of the prosthecate phenotype are expressed under oligotrophic conditions [52,54]. We are unaware of published reports on the metabolic interaction of the *Caulobacter*-like bacteria with other members of microbial communities [52]. However, it is believed that the adhesion of these bacteria to the algal cells might be important for the enrichment of the phycosphere with bioavailable organic matter [53]. In addition, the autotrophic cells are the source of O_2_ under illuminated conditions, which is the most attractive factor for *Caulobacter*-like bacteria occurring in algal cultures [55]. At the same time, according to Hentchel et al. [53], excessively high O_2_ levels can be toxic for the bacterium. Therefore, taking into account the decline in photosynthesis and corresponding photoproduction of O_2_ [17,30] and thermal dissipation of absorbed light energy in *H. lacustris* cells [17] under the inductive conditions, the coexistence of *Haematococcus* and *Caulobacter*-like bacteria is more likely to occur during the stress-induced astaxanthin accumulation.

### 3.3. Changes in H. lacustris Bacterial Community Composition during Astaxanthin Accumulation

Judging from the values of the predicted abundance estimators at the genus (Table S1) or family (Table S2) level, the obtained datasets almost completely described the bacterial diversity in the samples. Based on *H* values (Figure 2E), the induction of astaxanthin synthesis by stress was accompanied by a gradual decline in the bacterial diversity. This trend was not reversed during the recovery of vegetative growth; the values of *d* changed in a similar manner (Figure 2E). These changes in biodiversity can be explained by increased competitiveness and hence domination capacity of different bacterial taxa under the stress used for the induction of carotenogenesis.

The values of the Morisita–Horn dissimilarity index (*χ_ij_*) calculated for each pair of the samples *i* and *j≠i* at the genus level are presented on the Figure 2F. The values of the index closer to one (red highlight on the heatmap) reflect the high degree of dissimilarity of bacterial communities from a pair of samples in terms of the presence and abundance of different genera. By contrast, the values of *χ_ij_* close to zero reflect a high degree of similarity in the community taxonomic structure (blue highlight). The sample from the culture before the astaxanthin induction was characterized by high *χ_ij_* values in all sample pairs. In other words, the formal β-diversity analysis (Figure 2F) showed that the bacterial community of the *H. lacustris* vegetative cell culture differed strongly from that of the stressed cultures. Less pronounced changes were observed between the samples taken at different time points of *H. lacustris* exposure to stress (during the inductive stage; Figure 2F). The values of *χ_ij_* between samples from stress cultures at 2–4 days were lowest. Thus, these samples were very similar in terms of their bacterial community taxonomical structure. At the same time, the pairs of samples collected under stress and after recovery were characterized by moderate *χ_ij_* values (white color on the heatmap, Figure 2F). This means that the short term recovery also shifted the community established under the stress conditions but these changes were not as sharp as in the case of the astaxanthin synthesis induction in the vegetative culture. Thus, the exposure of *H. lacustris* cultures to the stress inducing accumulation of astaxanthin brought about a sharp, irreversible (at least on the timescale of our experiment) change in the structure of the bacterial community formed around the microalgal cell. On the contrary, the conditions conductive for the vegetative growth exerted a stabilizing effect on the bacterial community.

**Figure 3:**
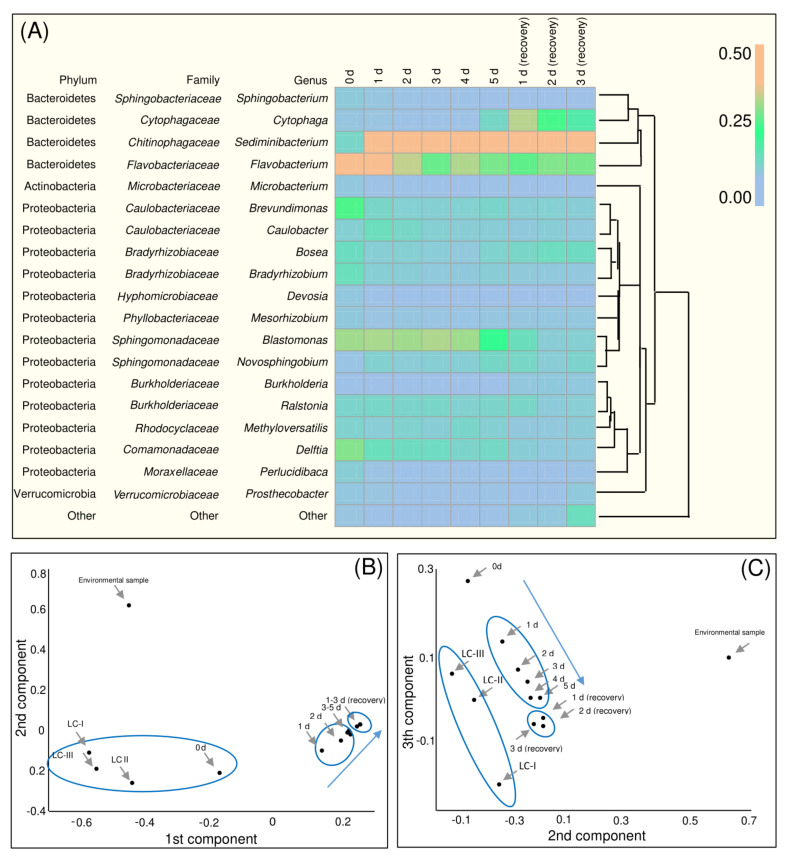
(**A**) Heatmap of bacterial taxa abundant in the samples of *H. lacustris* associations painted based on the fraction of NGS reads corresponding to each genus. Color scale corresponds to fraction of reads. The taxa with NGS reads fraction < 1% were not considered. The phylogenetic relationship of taxa was reconstructed by a neighborhood joining algorithm. The results of the principal component analysis (PCA) based on the matrix of Morisita–Horn indices for each sample pairs (**B**) projection of the space of three components on the plain of the 1st and 2nd components and (**C**) on the plain of 2nd and 3rd components. LC-I, LC-II, LC-III (the laboratory *H. lacustris* cultures maintained after isolation from environment, see Material and Methods) and ‘environment sample’ are datasets from [26]. Blue arrows reflect the experiment time.

The list of taxa encountered in the studied samples is presented in Table S3. The bacterial taxa discovered in *H. lacustris* culture from the photobioreactor (Table S3) were classified according to the 131–204 genera, most of which were represented by ‘trace’ amounts of NGS reads (<1% of total number of reads); only 21 genera were represented by a sizeable number of reads (≥1% of total; Figure 3A). The bacteria found in the samples were represented by two phyla, Proteobacteria and Bacteroidetes; a very small fraction of Actinobacteria was presented (Figure 2G).

In general, *H. lacustris* cultivated in a photobioreactor was characterized by a lower bacterial abundance (in terms of the total number of the observed taxa) than the natural communities sampled from nature [26]. Only three phyla were discovered in the photobioreactor grown cultures versus up to eight phyla in the bacterial communities from the environmental samples.

Under stressful conditions, the fraction of reads corresponding to Bacteroidetes increased whereas under the conditions promoting the recovery of vegetative growth it decreased (Figure 2G). The ample presence of Bacteroidetes is a common feature of natural algal consortia samples and laboratory communities [56,57,58,59]. Their mass development is observed during algal blooms [59], most likely due to possessing a wide range of lytic enzymes allowing them to feed on algal cell exopolymers [59]. Collectively, our data confirmed previous findings showing that Proteobacteria and Bacteroidetes are the common phyla of bacteria accompanying microalgal cultures from different natural and artificial habitats [60,61].

Cultures of *H. lacustris* from bioreactors were characterized by a high fraction of bacteria from the *Cytophaga-Flavobacterium* [62] cluster, i.e., genera *Cytophaga* and *Flavobacterium* (Figure 3A). In natural habitats, bacteria of this group are associated with marine phytoplankton [57,60,62]; they promote algal cell lysis and produce assorted polymer-decomposing hydrolases [59,62,63]. These bacteria were also found in the environmental samples but were less abundant [26]. The abundance of *Cytophaga* associated reads increased on the final day of stress exposure and remained relatively high after vegetative growth recovery.

Representatives of *Flavobacterium* are also common for marine phytoplankton and are capable of degrading biopolymers [57,62]. Notably, these bacteria, typical for marine environments [62], were abundant in the laboratory BG-11 medium with zero salinity. Representatives of *Flavobacterium* were also abundant in the *H. lacustris* vegetative cell culture but their amount decreased after exposure of the culture to stress (Figure 3A). This was in accordance with previous data on *H. lacustris* laboratory cultures [64] and natural communities [26]. Similarly to *Flavobacterium*, the number of *Delftia* reads decreased after stress exposure (Figure 3A). *Delftia* is abundant in cultures of green microalgae [65]. This bacterium has been reported as capable of promoting plant growth [66]. It may be involved in the regulation of microalgae growth as well. The bacteroidete *Sediminibacterium* became the most abundant (in terms of NGS read fraction size) bacterium in the cultures subjected to the stressful conditions. Previously it was found in laboratory and industrial cultures of *Chlamydomonas* (Chlorophyceae), *Botryococcus* (Trebouxiophyceae), *Nannochloropsis* (Eustigmatophyceae) microalgae [61], *Chlorella* (Trebouxiophyceae) and *Dunaliella* (Chlorophyceae) [67]. A relatively high fraction of *Blastomonas* from the family *Sphingomonadaceae* was observed until the recovery stage (Figure 3A). It was previously isolated from the *H. lacustris* laboratory cultures [64]. The presence of this photoheterotrophic strictly aerobic taxa probably was due to the light regime in the photobioreactor and O_2_ production by the microalgae. *Novosphingobium*, another moderately abundant *Sphingomonadaceae* bacterium, is known to promote plant growth [68]. *Burkholderiaceae Ralstonia* was another moderately abundant bacterium (Figure 3A). Its presence has been noted in microalgal cultures [67,69]. Moreover, it has also been isolated from laboratory cultures of *H. lacustris* [64]. For two representatives of *Bradyrhizobiaceae*, *Bosea* and *Bradyrhizobium*, relatively high fractions of NGS reads were observed in the samples before the astaxanthin synthesis induction (Figure 3A). *Bosea* was also abundant in the *H. lacustris* laboratory cultures but not in the environmental samples [26]. *Bradyrhizobium* was previously found in laboratory cultures of other chlorophytes (*Botryococcus* [69] and *Chlorella* [70]). *Caulobacteriaceae* (especially *Caulobacter*) was observed in the *H. lacustris* culture (Figure 3A). The later finding was in accordance with the microscopic observations. Therefore, these bacteria might be considered as a component of the *H. lacustris* phycosphere in photobioreactors. Another representative of this family, *Brevundimonas*, which was found in photobioreactor-cultivated *H. lacustris* especially under vegetative growth conditions (Figure 3A), also presented in a few environmental samples of this microalga from the White Sea coast [26].

Previously we discovered *Comamonadaceae*, *Cytophagaceae*, *Xanthomonadaceae*, *Acetobacteraceae*, *Rhodobacteraceae* and *Rhodocyclaceae* in the natural microbial communities formed around *H. lacustris* from the White Sea coastal rock baths [26]. Under laboratory conditions, only *Comamonadaceae*, *Cytophagaceae* and *Rhodobacteraceae* retained a relatively high abundance (Figure 3A). In addition, *Hydrogenophaga*, which was common for *H. lacustris*-containing environmental samples and the isolates of this microalga [26], also was revealed in the *H. lacustris* cultures throughout the experiment although the fraction size of its reads was small (Table S3).

Previous works have contributed to understanding whether a species-specific composition of the microalgal phycosphere exists. *Flavobacterium, Rhizobium, Sphingomonas, Sphingobium* and *Sediminibacterium* were found in cultures of chlorophytes [60,61]. *Roseobacteriaceae* were also represented in algal cultures [60]. These facts may reflect a possible interaction of these bacteria with microalgae. Many of their representatives belong to the Plant Growth Promoting Bacteria (PGPB; [70,71]), the group of bacteria feeding on algal exopolymers [61] and excreting bioactive substances, such as phytohormones, promoting algal growth. As a result, the PGPBs enhance microalgal cell division and vice versa: the coexistence with microalgae promotes the growth of PGPB [70].

To assess the influence of external microbiota on *H. lacustris* cultures in photobioreactors we obtained a bacterial composition of the laboratory where microalgae were cultured. The bacteriome of the laboratory was characterized by the predominance of five phyla: Proteobacteria, Actinobacteria, Clsotridia, Bacilli and Sphingobacteria. Proteobacteria and Actinobacteria were most abundant (S4). A comparison of the bacterial composition in the laboratory and in the photobioreactor-cultivated *H. lacustris* cultures by *χ_ij_* calculation at the genus level showed a significant dissimilarity between their taxonomical structures (S4). The values of *χ_ij_* between the external samples from the laboratory and samples from the *H. lacustris* cultures were close to one whereas *χ_ij_* values between the samples from the photobioreactor-cultivated *H. lacustris* were in the range of 0.01–0.15. This was confirmed by the PCA (S4): the point corresponding to the external sample from the laboratory was far from the cluster of points corresponding to the samples from the *H. lacustris* cultures. The most abundant in the external laboratory sample were Actinobacteria *Micrococcus*, *Propionibacterium*, *Corynebacterium* and *Rothia* and Bacilli *Macrococcus*, *Staphylococcus* and *Streptococcus*, members of *Vibropnaceae*, *Moraxellaceae* and *Comamonadaceae* (S4). It should be noted that small fractions (<1% of NGS reads in the samples) of *Brevundimonas*, *Bradyrhizobium* and *Bosea* presented in the photobioreactor-cultivated *H. lacustris* also were detected. Collectively, one can conclude that the external microbial community did not significantly affect the bacterial composition of photobioreactor-cultivated *H. lacustris* because there were different main bacterial groups and representatives. However, external microbiota might permeate the photobioreactor due to the non-absolute sterility of semi-industrial and industrial systems. It might be seen as the increasing of a small fraction of Actinobacteria (abundant in the external laboratory samples) in the *H. lacustris* cultures. Therefore, a small actinobacterial fraction might be considered as a contamination.

A PCA based on the *χ_ij_* values calculated at the genus level revealed the difference in the taxonomical structures of the bacterial community through the samples of the *H. lacustris* cultures. In the *χ_ij_*-based three-component space, NGS reads datasets of the samples from *H. lacustris* bacterial communities were broken up into several clusters (Figure 3B,C). The datasets of the samples from the *H. lacustris* culture under the vegetative growth conditions in a photobioreactor clustered with control laboratory cultures from [34] on the projection of the principal components’ space to the cross-section of the 1st and 2nd components (Figure 3B). On the projection on the plane of the 2nd and 3rd components it was located separately (Figure 3C). Points corresponding to the states of the *H. lacustris* community after transferring to the astaxanthin accumulating conditions formed their own cluster (Figure 3B,C). Notably, moving the points in the principal component space reflected the dynamic of the *H. lacustris* bacterial community changing (see arrows on Figure 3B,C), forming extended clusters. The points corresponding to the community after vegetative growth recovery are located separately near to each other and near the cluster of the states under the conditions of astaxanthin accumulation (Figure 3B,C). Thus, despite *H. lacustris* being characterized by a fast recovery of photosynthetic activity and division rate [16], changes in the bacterial community composition seem to be irreversible, at least at short cultivation times. Based on the PCA and the dynamic of diversity indices (*H* and *d*), the conditions transferring to astaxanthin accumulation autotrophic conditions modulated the composition of the *H. lacustris* bacterial community in a certain direction. At the same time, vegetative growth conditions had a stabilizing effect on the bacterial component of the community. The states corresponding to the samples collected after growth recovery formed a distinct condensed cluster on the PC plots (Figure 3). Collectively, a *χ_ij_*-based PCA might reflect the shaping of the bacterial community in the *H. lacustris* from photobioreactors by evolved culturing conditions. 

## 4. Conclusions

As far as the authors know, this work is the first report on the bacteriome of *H. lacustris* cultivated in a photobioreactor. These findings are of potential significance for biotechnology. On one hand, they provide an insight into the possible bacterial contamination of harvested algal biomass. On the other hand, they reveal the presence of bacteria essential for the growth of the carotenogenic microalgae especially the bacteria from the PGPB group. The use of the algal-bacterial consortia enriched with the compatible PGPB represent an attractive (but largely underexplored) avenue of enhancing the productivity of natural astaxanthin producer microalgae.

The isolation of *H. lacustris* and its subsequent maintenance under laboratory conditions was accompanied by the depletion of the bacterial diversity in the cultures. In contrast to environmental samples of the microalga, it contained neither soil spore-forming bacteria nor cyanobacteria bacteria; the marine bacteria were also missing. Only traces of gram-positive bacteria were found. An important finding was the lack of pathogenic bacteria in the biomass of the microalga grown in the photobioreactor.

A decline in bacterial diversity was promoted by the exposure of the microalgal cultures to stressful conditions. This important finding suggests that the astaxanthin-rich biomass of *H. lacustris* is expected to contain a lower number of diverse bacteria than the biomass with a small astaxanthin content.

Collectively, our data suggest that *H. lacustris* autotrophically grown in a photobioreactor normally exists in the form of a consortium dominated by gram-negative bacteria from the phyla Proteobacteria or Bacteroidetes. Considering these groups of bacteria as members of the core *H. lacustris* microbiome needs further confirmation from studies with different laboratory and production cultures carried out with different algal strains. Of separate interest for future research are the possible mutualistic relationships of carotenogenic microalgae such as *H. lacustris* in the laboratory and production scale cultivation systems.

## Figures and Tables

**Table 1:** Description of laboratory *Haematococcus lacustris* cultures taken as a control for the analysis.

Culture Abbreviation	*H. lacustris* Strain	GenBank ID
LC-I	BMP/16	MH188841.1
LC-II	BMK/16	MH191369.1
LC-III	BMM1/16	MH188837.1

## Supplementary Materials

The following are available online at https://www.mdpi.com/2079-7737/10/2/115/s1, Table S1. The parameters of α-diversity calculated at a genus level for the samples collected each day during the astaxanthin synthesis induction in the *H. lacustris* BM1 (IPPAS H-2018) culture as well as during the recovery of the culture under the vegetative growth conditions: ACE (N_ACE_ and Chao-1 (N_Chao-1_) abundance estimators, Shannon entropy index (*H*) and reverse Simpson index (*d*). The threshold is > 1% of NGS reads. Table S2. The parameters of α-diversity calculated at a family level for the samples collected each day during the astaxanthin synthesis induction in the *H. lacustris* BM1 (IPPAS H-2018) culture as well as during the recovery of the culture under the vegetative growth conditions: ACE (N_ACE_ and Chao-1 (N_Chao-1_) abundance estimators, Shannon entropy index (*H*) and reverse Simpson index (*d*). The threshold is > 1% of NGS reads. Table S3. Total list of bacterial taxa presenting in *H. lacustris* BM1 (IPPAS H-2018) cultures during astaxanthin accumulation and vegetative growth recovery. The percentages of NGS reads corresponding to taxa from the total number of reads in the samples. File S4. The parameters of the laboratory external bacterial community.
