# Supplementary material for: The Dynamics of the Bacterial Community of the Photobioreactor-Cultivated Green Microalga Haematococcus lacustris during Stress-Induced Astaxanthin Accumulation
Source: Biology (Basel). 2021 Feb 4;10(2):115. doi: 10.3390/biology10020115 (PMC7915213; doi:10.3390/biology10020115)
Supplement: Supplementary file 1 [file biology-10-00115-s001.zip › Supplementary/S4.pdf]

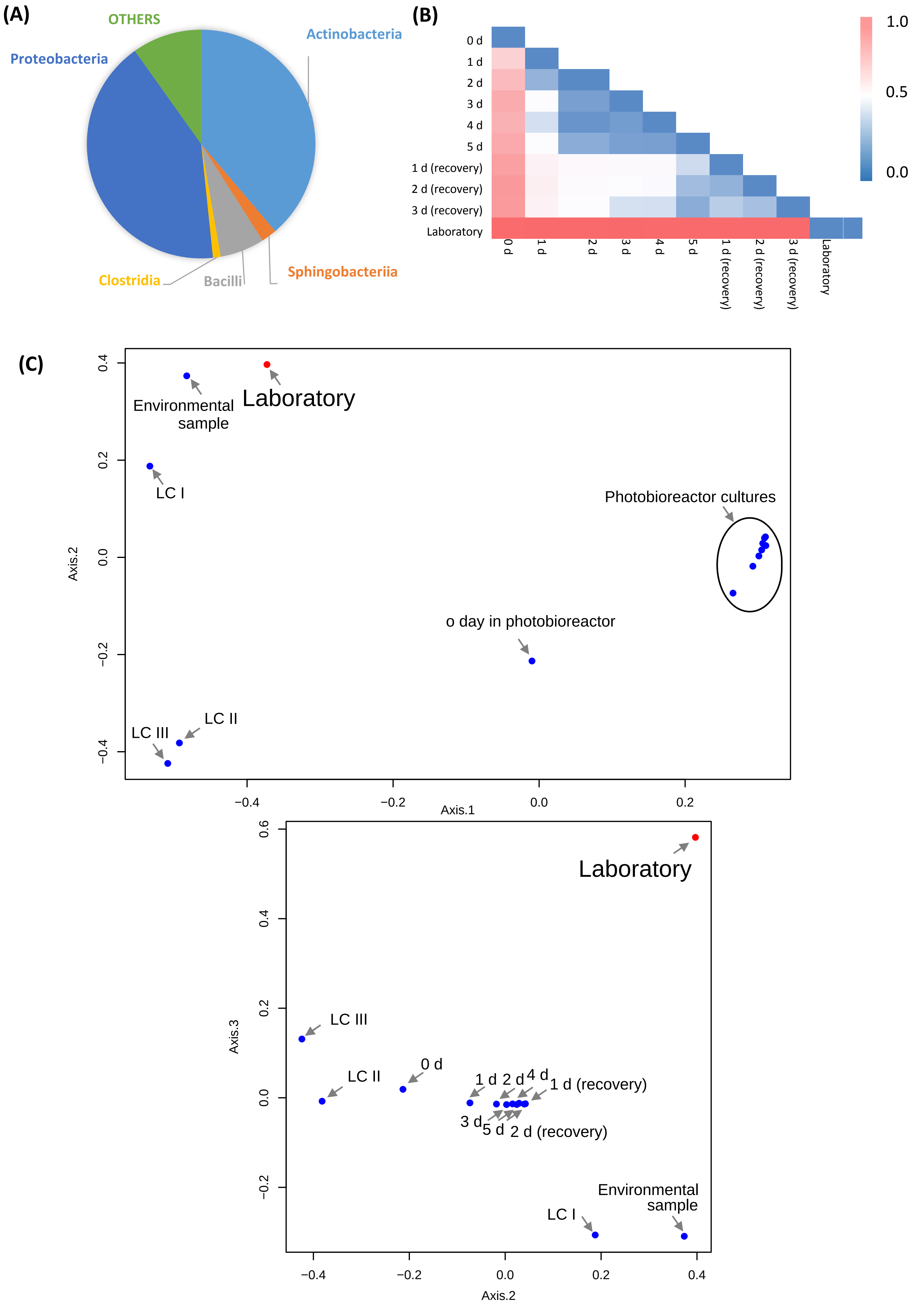

(A)
OTHERS
Actinobacteria
Proteobacteria
Clostridia
Bacilli
Sphingobacteriia
(B)
0 d
1 d
2 d
3 d
4 d
5 d
1 d (recovery)
2 d (recovery)
3 d (recovery)
Laboratory
1.0
0.5
0.0
(C)
Environmental sample
Laboratory
LC I
Photobioreactor cultures
o day in photobioreactor
LC II
LC III
Axis.1
Axis.2
Axis.3
0 d
1 d
2 d
4 d
1 d (recovery)
3 d
5 d
2 d (recovery)

Fig. S4. Taxonomical composition of bacterial community at the level of phylum in the laboratory, where *H. lacustris* BM1 was cultured **(A)**. The heatmap of the Morisita-Horn index values at the genus level reflecting similarity of the *16SrRNA* gene libraries obtained for the *H. lacustris*-bacterial association at different stages of the experiment, as well as with the dataset from the laboratory **(B)**. projection of the space of three components on the plain of 1st and 2nd components and on the plain of 2nd and 3th components. Samples from photobioreactor, LC-I, LC-II, LC-III (the laboratory *H. lacustris* cultures maintained after isolation), 'environment sample' as well as laboratory sample are presented **(C)**.

Table S4. Total list of bacterial genera observed in the laboratory, where *H. lacustris* BM1 (IPPAS H-2018) was cultured based on sequencing of *16SrRNA* gene fragment. The percentages of total bacterial reads in each are presented.

Phylum	Family	Genus	Reads percentage
Actinobacteria	*Corynebacteriaceae*	*Corynebacterium*	1.24
	Micrococcaceae	*Micrococcus*	3.49
		Rothia	1.08
	Propionibacteriaceae	*Propionibacterium*	28.52
	OTHERS		4.45
Sphingobacteriia	OTHERS		2.20
Bacilli	*Staphylococcaceae*	*Macrococcus*	3.48
		Staphylococcus	1.42
	Streptococcaceae	*Streptococcus*	1.38
Clostridia	*Ruminococcaceae*	*Ruminococcus*	0.31
		Subdoligranulum	0.47
		OTHERS	0.31
Proteobacteria	*Caulobacteraceae*	*Brevundimonas*	0.78
	Bradyrhizobiaceae	*Bosea*	0.28
		Bradyrhizobium	0.47
	Phyllobacteriaceae	*Mesorhizobium*	0.45
	Rhizobiaceae	*Ensifer*	2.53
	Alcaligenaceae	*Achromobacter*	0.04
	Comamonadaceae	OTHERS	2.28
	Pasteurellaceae	*Haemophilus*	1.30
	Moraxellaceae	*Acinetobacter*	3.62
		Enhydrobacter	1.19
		Psychrobacter	1.11
	Pseudomonadaceae	*Pseudomonas*	2.74
	Vibrionaceae	OTHERS	17.98
	Xanthomonadaceae	OTHERS	0.91
	OTHERS		6.12
OTHERS			9.85
