# Supplementary material for: The Dynamics of the Bacterial Community of the Photobioreactor-Cultivated Green Microalga Haematococcus lacustris during Stress-Induced Astaxanthin Accumulation
Source: Biology (Basel). 2021 Feb 4;10(2):115. doi: 10.3390/biology10020115 (PMC7915213; doi:10.3390/biology10020115)
Supplement: Supplementary file 1 [file biology-10-00115-s001.zip › Supplementary/Table s1.docx]

| **Sample** | **Observed richness** | **N_ACE_** | **N_Chao-1_** | ***H*** | ***d*** |  |
| --- | --- | --- | --- | --- | --- | --- |
| 0 d | 20 | 20 | 20 | 3.36 | 0.86 |  |
| 1 d | 20 | 20 | 20 | 2.99 | 0.81 |  |
| 2 d | 20 | 20 | 20 | 2.74 | 0.76 |  |
| 3 d | 20 | 20 | 20 | 2.57 | 0.72 |  |
| 4 d | 20 | 20 | 20 | 2.56 | 0.72 |  |
| 5 d | 20 | 20 | 20 | 2.79 | 0.74 |  |
| 1 d (recovery) | 20 | 20 | 20 | 2.80 | 0.76 |  |
| 2 d (recovery) | 20 | 20 | 20 | 2.54 | 0.70 |  |
| 3 d (recovery) | 20 | 20 | 20 | 2.63 | 0.69 |  |

**Table S1.** The parameters of α-diversity calculated at genus level for the samples collected each day during astaxanthin synthesis induction in the *H. lacustris* BM1 (IPPAS H-2018) culture as well as during recovery of the culture under the vegetative growth conditions: ACE (N_ACE_ and Chao-1 (N_Chao-1_) abundance estimators, Shannon entropy index (*H*), and reverse Simpson index (*d*). The taxa with the abundance lower than 1% reads were not taken into account.
