# Supplementary material for: The Dynamics of the Bacterial Community of the Photobioreactor-Cultivated Green Microalga Haematococcus lacustris during Stress-Induced Astaxanthin Accumulation
Source: Biology (Basel). 2021 Feb 4;10(2):115. doi: 10.3390/biology10020115 (PMC7915213; doi:10.3390/biology10020115)
Supplement: Supplementary file 1 [file biology-10-00115-s001.zip › Supplementary/Table s3.docx]

**Table S3.** Total list of bacterial genera observed in the samples for the samples collected each day during astaxanthin synthesis induction in the *H. lacustris* BM1 (IPPAS H-2018) culture as well as during recovery of the culture under the vegetative growth conditions. The percentages of total bacterial reads in each are presented.

| **Phylum** | **Family** | **Genus** | **0 d** | **1 d** | **2 d** | **3 d** | **4 d** | **5 d** | **1 d recovery** | **2 d recovery** | **3 d recovery** |
| --- | --- | --- | --- | --- | --- | --- | --- | --- | --- | --- | --- |
| Acidobacteria | empty_family | *Blastocatella* | 0.00 | 0.00 | 0.00 | 0.00 | 0.00 | 0.00 | 0.00 | 0.01 | 0.00 |
| Acidobacteria | empty_family | *Bryobacter* | 0.00 | 0.00 | 0.00 | 0.00 | 0.00 | 0.00 | 0.00 | 0.00 | 0.01 |
| Acidobacteria | family_NA | *genus_NA* | 0.00 | 0.01 | 0.00 | 0.00 | 0.00 | 0.00 | 0.00 | 0.00 | 0.02 |
| Actinobacteria | family_NA | *genus_NA* | 0.07 | 0.00 | 0.00 | 0.00 | 0.00 | 0.00 | 0.00 | 0.00 | 0.00 |
| Actinobacteria | Bifidobacteriaceae | *Gardnerella* | 0.00 | 0.01 | 0.00 | 0.00 | 0.00 | 0.00 | 0.00 | 0.00 | 0.00 |
| Actinobacteria | Corynebacteriaceae | *Corynebacterium* | 0.02 | 0.00 | 0.00 | 0.00 | 0.00 | 0.00 | 0.00 | 0.00 | 0.00 |
| Actinobacteria | Dietziaceae | *Dietzia* | 0.00 | 0.00 | 0.00 | 0.00 | 0.00 | 0.01 | 0.00 | 0.00 | 0.00 |
| Actinobacteria | Mycobacteriaceae | *Mycobacterium* | 0.37 | 0.08 | 0.12 | 0.03 | 0.03 | 0.04 | 0.03 | 0.03 | 0.06 |
| Actinobacteria | Nocardiaceae | *Rhodococcus* | 0.24 | 0.00 | 0.00 | 0.01 | 0.00 | 0.00 | 0.00 | 0.00 | 0.00 |
| Actinobacteria | Nakamurellaceae | *Nakamurella* | 0.00 | 0.00 | 0.00 | 0.00 | 0.00 | 0.00 | 0.00 | 0.00 | 0.01 |
| Actinobacteria | Sporichthyaceae | *genus_NA* | 0.00 | 0.00 | 0.00 | 0.00 | 0.00 | 0.00 | 0.00 | 0.00 | 0.02 |
| Actinobacteria | Cellulomonadaceae | *Cellulomonas* | 0.14 | 0.00 | 0.01 | 0.02 | 0.00 | 0.01 | 0.01 | 0.05 | 0.00 |
| Actinobacteria | Dermatophilaceae | *Dermatophilus* | 0.00 | 0.00 | 0.00 | 0.00 | 0.00 | 0.00 | 0.00 | 0.01 | 0.00 |
| Actinobacteria | Dermatophilaceae | *Kineosphaera* | 0.00 | 0.00 | 0.00 | 0.00 | 0.01 | 0.00 | 0.00 | 0.00 | 0.00 |
| Actinobacteria | Microbacteriaceae | *Leifsonia* | 0.01 | 0.01 | 0.01 | 0.02 | 0.01 | 0.01 | 0.01 | 0.00 | 0.00 |
| Actinobacteria | Microbacteriaceae | *Microbacterium* | 1.06 | 0.26 | 0.27 | 0.25 | 0.17 | 0.31 | 0.10 | 0.05 | 0.09 |
| Actinobacteria | Microbacteriaceae | *Mycetocola* | 0.01 | 0.01 | 0.00 | 0.00 | 0.00 | 0.02 | 0.01 | 0.01 | 0.01 |
| Actinobacteria | Microbacteriaceae | *Plantibacter* | 0.05 | 0.01 | 0.03 | 0.01 | 0.01 | 0.02 | 0.01 | 0.00 | 0.00 |
| Actinobacteria | Microbacteriaceae | *Rathayibacter* | 0.01 | 0.01 | 0.02 | 0.00 | 0.00 | 0.00 | 0.00 | 0.00 | 0.00 |
| Actinobacteria | Microbacteriaceae | *genus_NA* | 0.05 | 0.01 | 0.01 | 0.00 | 0.01 | 0.01 | 0.00 | 0.00 | 0.00 |
| Actinobacteria | Micrococcaceae | *Micrococcus* | 0.01 | 0.00 | 0.00 | 0.00 | 0.00 | 0.00 | 0.00 | 0.00 | 0.00 |
| Actinobacteria | family_NA | *genus_NA* | 0.01 | 0.00 | 0.00 | 0.00 | 0.00 | 0.00 | 0.00 | 0.00 | 0.00 |
| Actinobacteria | Propionibacteriaceae | *Propionibacterium* | 0.09 | 0.01 | 0.01 | 0.02 | 0.02 | 0.01 | 0.01 | 0.01 | 0.02 |
| Actinobacteria | Pseudonocardiaceae | *Pseudonocardia* | 0.00 | 0.00 | 0.01 | 0.00 | 0.00 | 0.00 | 0.00 | 0.00 | 0.00 |
| Actinobacteria | Thermomonosporaceae | *Actinocorallia* | 0.00 | 0.01 | 0.00 | 0.00 | 0.00 | 0.00 | 0.00 | 0.00 | 0.00 |
| Actinobacteria | Coriobacteriaceae | *genus_NA* | 0.00 | 0.00 | 0.00 | 0.00 | 0.00 | 0.00 | 0.00 | 0.00 | 0.01 |
| Actinobacteria | family_NA | *genus_NA* | 0.00 | 0.00 | 0.00 | 0.00 | 0.00 | 0.00 | 0.00 | 0.00 | 0.01 |
| Actinobacteria | Patulibacteraceae | *Patulibacter* | 0.00 | 0.00 | 0.00 | 0.00 | 0.00 | 0.00 | 0.00 | 0.00 | 0.00 |
| Actinobacteria | family_NA | *genus_NA* | 0.01 | 0.00 | 0.00 | 0.00 | 0.00 | 0.00 | 0.00 | 0.00 | 0.00 |
| Armatimonadetes | family_NA | *genus_NA* | 0.00 | 0.00 | 0.00 | 0.01 | 0.00 | 0.00 | 0.00 | 0.00 | 0.00 |
| Armatimonadetes | family_NA | *genus_NA* | 0.00 | 0.00 | 0.01 | 0.00 | 0.00 | 0.00 | 0.00 | 0.00 | 0.00 |
| Bacteroidetes | Cytophagaceae | *Cytophaga* | 0.82 | 0.66 | 0.29 | 0.26 | 0.33 | 3.48 | 16.04 | 10.72 | 6.66 |
| Bacteroidetes | Cytophagaceae | *Hymenobacter* | 0.00 | 0.00 | 0.02 | 0.01 | 0.01 | 0.00 | 0.01 | 0.04 | 0.01 |
| Bacteroidetes | Cytophagaceae | *Larkinella* | 0.02 | 0.00 | 0.00 | 0.00 | 0.00 | 0.00 | 0.00 | 0.00 | 0.02 |
| Bacteroidetes | Cytophagaceae | *Persicitalea* | 0.00 | 0.00 | 0.00 | 0.00 | 0.01 | 0.00 | 0.00 | 0.00 | 0.00 |
| Bacteroidetes | Cytophagaceae | *Pontibacter* | 0.00 | 0.00 | 0.00 | 0.01 | 0.00 | 0.01 | 0.00 | 0.00 | 0.00 |
| Bacteroidetes | Cytophagaceae | *Spirosoma* | 0.00 | 0.00 | 0.04 | 0.03 | 0.02 | 0.00 | 0.04 | 0.02 | 0.02 |
| Bacteroidetes | Cytophagaceae | *Sporocytophaga* | 0.00 | 0.00 | 0.00 | 0.00 | 0.00 | 0.00 | 0.02 | 0.03 | 0.01 |
| Bacteroidetes | Cytophagaceae | *genus_NA* | 0.02 | 0.00 | 0.00 | 0.00 | 0.00 | 0.00 | 0.01 | 0.03 | 0.01 |
| Bacteroidetes | family_NA | *genus_NA* | 0.00 | 0.00 | 0.00 | 0.00 | 0.00 | 0.00 | 0.00 | 0.01 | 0.00 |
| Bacteroidetes | Flavobacteriaceae | *Aequorivita* | 0.00 | 0.00 | 0.01 | 0.00 | 0.00 | 0.00 | 0.00 | 0.00 | 0.00 |
| Bacteroidetes | Flavobacteriaceae | *Chryseobacterium* | 0.00 | 0.00 | 0.00 | 0.00 | 0.00 | 0.00 | 0.00 | 0.00 | 0.01 |
| Bacteroidetes | Flavobacteriaceae | *Flavobacterium* | 24.88 | 20.09 | 16.29 | 12.34 | 15.62 | 13.03 | 11.85 | 13.43 | 13.69 |
| Bacteroidetes | Chitinophagaceae | *Ferruginibacter* | 0.02 | 0.04 | 0.03 | 0.04 | 0.05 | 0.05 | 0.01 | 0.01 | 0.05 |
| Bacteroidetes | Chitinophagaceae | *Hydrotalea* | 0.00 | 0.00 | 0.00 | 0.00 | 0.00 | 0.01 | 0.00 | 0.02 | 0.01 |
| Bacteroidetes | Chitinophagaceae | *Lacibacter* | 0.00 | 0.00 | 0.00 | 0.00 | 0.00 | 0.00 | 0.00 | 0.00 | 0.00 |
| Bacteroidetes | Chitinophagaceae | *Parasegetibacter* | 0.00 | 0.00 | 0.00 | 0.00 | 0.00 | 0.00 | 0.00 | 0.01 | 0.00 |
| Bacteroidetes | Chitinophagaceae | *Sediminibacterium* | 3.10 | 31.65 | 39.71 | 45.97 | 44.53 | 44.17 | 40.84 | 48.45 | 50.17 |
| Bacteroidetes | Chitinophagaceae | *Solibius* | 0.01 | 0.02 | 0.00 | 0.01 | 0.00 | 0.02 | 0.01 | 0.02 | 0.01 |
| Bacteroidetes | Chitinophagaceae | *genus_NA* | 0.00 | 0.04 | 0.05 | 0.02 | 0.03 | 0.03 | 0.05 | 0.07 | 0.08 |
| Bacteroidetes | Sphingobacteriaceae | *Mucilaginibacter* | 0.00 | 0.00 | 0.00 | 0.00 | 0.00 | 0.00 | 0.00 | 0.00 | 0.01 |
| Bacteroidetes | Sphingobacteriaceae | *Pedobacter* | 0.04 | 0.03 | 0.00 | 0.01 | 0.00 | 0.00 | 0.01 | 0.01 | 0.01 |
| Bacteroidetes | Sphingobacteriaceae | *Sphingobacterium* | 1.44 | 0.78 | 0.40 | 0.31 | 0.29 | 0.13 | 0.12 | 0.07 | 0.08 |
| Bacteroidetes | Sphingobacteriaceae | *genus_NA* | 0.00 | 0.00 | 0.00 | 0.00 | 0.00 | 0.00 | 0.01 | 0.00 | 0.00 |
| Bacteroidetes | family_NA | *genus_NA* | 0.18 | 0.17 | 0.13 | 0.10 | 0.07 | 0.14 | 0.33 | 0.10 | 0.20 |
| Bacteroidetes | family_NA | *genus_NA* | 0.02 | 0.00 | 0.01 | 0.01 | 0.01 | 0.00 | 0.01 | 0.01 | 0.01 |
| Chlorobi | family_NA | *genus_NA* | 0.00 | 0.01 | 0.00 | 0.00 | 0.00 | 0.00 | 0.00 | 0.00 | 0.00 |
| Chloroflexi | Anaerolineaceae | *genus_NA* | 0.00 | 0.00 | 0.00 | 0.00 | 0.00 | 0.00 | 0.00 | 0.00 | 0.01 |
| Chloroflexi | Caldilineaceae | *genus_NA* | 0.00 | 0.00 | 0.01 | 0.00 | 0.00 | 0.00 | 0.00 | 0.00 | 0.01 |
| Chloroflexi | family_NA | *genus_NA* | 0.00 | 0.01 | 0.00 | 0.00 | 0.00 | 0.00 | 0.00 | 0.00 | 0.00 |
| Chloroflexi | family_NA | *genus_NA* | 0.00 | 0.01 | 0.00 | 0.00 | 0.00 | 0.00 | 0.00 | 0.00 | 0.00 |
| Chloroflexi | family_NA | *genus_NA* | 0.00 | 0.00 | 0.00 | 0.00 | 0.00 | 0.00 | 0.00 | 0.01 | 0.00 |
| Cyanobacteria | empty_family | *Microcystis* | 0.00 | 0.00 | 0.00 | 0.00 | 0.00 | 0.00 | 0.00 | 0.00 | 0.01 |
| Cyanobacteria | Nostocaceae | *Nostoc* | 0.00 | 0.00 | 0.00 | 0.01 | 0.00 | 0.00 | 0.00 | 0.00 | 0.00 |
| Cyanobacteria | Rivulariaceae | *Rivularia* | 0.00 | 0.00 | 0.00 | 0.00 | 0.00 | 0.00 | 0.00 | 0.04 | 0.00 |
| Cyanobacteria | empty_family | *Leptolyngbya* | 0.00 | 0.00 | 0.06 | 0.01 | 0.04 | 0.00 | 0.01 | 0.07 | 0.13 |
| Cyanobacteria | empty_family | *Microcoleus* | 0.00 | 0.01 | 0.19 | 0.02 | 0.04 | 0.00 | 0.03 | 0.04 | 0.01 |
| Cyanobacteria | empty_family | *Oscillatoria* | 0.00 | 0.00 | 0.00 | 0.00 | 0.00 | 0.00 | 0.00 | 0.02 | 0.00 |
| Cyanobacteria | empty_family | *Phormidium* | 0.01 | 0.01 | 0.14 | 0.02 | 0.08 | 0.01 | 0.01 | 0.31 | 0.00 |
| Cyanobacteria | empty_family | *Pleurocapsa* | 0.00 | 0.00 | 0.00 | 0.00 | 0.00 | 0.01 | 0.00 | 0.00 | 0.00 |
| Cyanobacteria | family_NA | *genus_NA* | 0.00 | 0.00 | 0.00 | 0.00 | 0.00 | 0.00 | 0.00 | 0.00 | 0.01 |
| Cyanobacteria | family_NA | *genus_NA* | 0.01 | 0.00 | 0.03 | 0.01 | 0.01 | 0.00 | 0.01 | 0.01 | 0.02 |
| Firmicutes | Bacillaceae | *Bacillus* | 0.01 | 0.01 | 0.00 | 0.00 | 0.00 | 0.01 | 0.00 | 0.00 | 0.01 |
| Firmicutes | Bacillaceae | *Exiguobacterium* | 0.00 | 0.00 | 0.00 | 0.00 | 0.02 | 0.02 | 0.00 | 0.00 | 0.01 |
| Firmicutes | Paenibacillaceae | *Paenibacillus* | 0.29 | 0.08 | 0.07 | 0.03 | 0.04 | 0.03 | 0.04 | 0.01 | 0.05 |
| Firmicutes | Staphylococcaceae | *Macrococcus* | 0.03 | 0.00 | 0.00 | 0.01 | 0.00 | 0.00 | 0.00 | 0.01 | 0.02 |
| Firmicutes | Staphylococcaceae | *Staphylococcus* | 0.03 | 0.01 | 0.00 | 0.00 | 0.00 | 0.00 | 0.00 | 0.00 | 0.00 |
| Firmicutes | Streptococcaceae | *Streptococcus* | 0.00 | 0.00 | 0.00 | 0.01 | 0.00 | 0.00 | 0.00 | 0.00 | 0.00 |
| Firmicutes | Christensenellaceae | *genus_NA* | 0.00 | 0.00 | 0.00 | 0.00 | 0.00 | 0.00 | 0.00 | 0.00 | 0.01 |
| Firmicutes | Clostridiaceae_1 | *Clostridium_sensu_stricto_13* | 0.00 | 0.00 | 0.00 | 0.01 | 0.00 | 0.00 | 0.02 | 0.02 | 0.00 |
| Firmicutes | Lachnospiraceae | *genus_NA* | 0.00 | 0.00 | 0.00 | 0.00 | 0.00 | 0.00 | 0.01 | 0.00 | 0.00 |
| Firmicutes | Peptostreptococcaceae | *genus_NA* | 0.00 | 0.00 | 0.00 | 0.00 | 0.00 | 0.00 | 0.00 | 0.03 | 0.00 |
| Firmicutes | Ruminococcaceae | *Ruminococcus* | 0.00 | 0.00 | 0.00 | 0.00 | 0.00 | 0.01 | 0.00 | 0.00 | 0.00 |
| Firmicutes | Ruminococcaceae | *genus_NA* | 0.00 | 0.00 | 0.00 | 0.01 | 0.00 | 0.00 | 0.00 | 0.00 | 0.03 |
| Firmicutes | Erysipelotrichaceae | *genus_NA* | 0.00 | 0.00 | 0.00 | 0.00 | 0.00 | 0.00 | 0.00 | 0.00 | 0.01 |
| Proteobacteria | Caulobacteraceae | *Brevundimonas* | 10.36 | 3.33 | 2.39 | 2.31 | 2.50 | 3.12 | 2.12 | 2.35 | 2.04 |
| Proteobacteria | Caulobacteraceae | *Caulobacter* | 2.16 | 4.36 | 3.38 | 2.28 | 1.85 | 1.96 | 1.40 | 1.98 | 1.42 |
| Proteobacteria | Caulobacteraceae | *Phenylobacterium* | 0.10 | 0.31 | 0.29 | 0.28 | 0.77 | 0.77 | 0.39 | 0.39 | 0.35 |
| Proteobacteria | Caulobacteraceae | *genus_NA* | 0.01 | 0.00 | 0.00 | 0.01 | 0.00 | 0.00 | 0.00 | 0.02 | 0.00 |
| Proteobacteria | Beijerinckiaceae | *Methylocapsa* | 0.04 | 0.04 | 0.02 | 0.02 | 0.02 | 0.03 | 0.00 | 0.00 | 0.00 |
| Proteobacteria | Beijerinckiaceae | *Methyloferula* | 0.01 | 0.00 | 0.01 | 0.01 | 0.00 | 0.00 | 0.01 | 0.01 | 0.00 |
| Proteobacteria | Beijerinckiaceae | *genus_NA* | 0.02 | 0.00 | 0.01 | 0.01 | 0.01 | 0.02 | 0.01 | 0.02 | 0.00 |
| Proteobacteria | Bradyrhizobiaceae | *Afipia* | 0.10 | 0.03 | 0.03 | 0.02 | 0.02 | 0.03 | 0.05 | 0.03 | 0.01 |
| Proteobacteria | Bradyrhizobiaceae | *Bosea* | 4.00 | 2.01 | 2.18 | 1.78 | 1.32 | 2.68 | 3.35 | 4.22 | 4.17 |
| Proteobacteria | Bradyrhizobiaceae | *Bradyrhizobium* | 4.21 | 2.16 | 1.84 | 1.43 | 1.40 | 1.81 | 1.50 | 1.36 | 1.22 |
| Proteobacteria | Bradyrhizobiaceae | *Nitrobacter* | 0.13 | 0.07 | 0.04 | 0.03 | 0.02 | 0.04 | 0.03 | 0.05 | 0.02 |
| Proteobacteria | Bradyrhizobiaceae | *Rhodopseudomonas* | 0.04 | 0.00 | 0.00 | 0.01 | 0.00 | 0.02 | 0.01 | 0.04 | 0.01 |
| Proteobacteria | Bradyrhizobiaceae | *genus_NA* | 0.03 | 0.01 | 0.01 | 0.01 | 0.00 | 0.02 | 0.02 | 0.02 | 0.01 |
| Proteobacteria | Brucellaceae | *Ochrobactrum* | 0.00 | 0.00 | 0.00 | 0.00 | 0.00 | 0.01 | 0.00 | 0.00 | 0.00 |
| Proteobacteria | Hyphomicrobiaceae | *Devosia* | 1.12 | 0.24 | 0.13 | 0.13 | 0.09 | 0.14 | 0.12 | 0.08 | 0.05 |
| Proteobacteria | Hyphomicrobiaceae | *Rhodoplanes* | 0.01 | 0.01 | 0.00 | 0.01 | 0.00 | 0.01 | 0.00 | 0.01 | 0.01 |
| Proteobacteria | Hyphomicrobiaceae | *genus_NA* | 0.03 | 0.01 | 0.00 | 0.00 | 0.00 | 0.00 | 0.01 | 0.01 | 0.01 |
| Proteobacteria | Methylobacteriaceae | *Meganema* | 0.00 | 0.00 | 0.00 | 0.00 | 0.00 | 0.00 | 0.00 | 0.00 | 0.01 |
| Proteobacteria | Methylobacteriaceae | *Methylobacterium* | 0.84 | 0.22 | 0.25 | 0.18 | 0.13 | 0.47 | 0.57 | 1.00 | 0.79 |
| Proteobacteria | Phyllobacteriaceae | *Mesorhizobium* | 1.32 | 0.56 | 0.82 | 0.64 | 0.54 | 0.85 | 0.95 | 1.01 | 1.17 |
| Proteobacteria | Phyllobacteriaceae | *Nitratireductor* | 0.00 | 0.01 | 0.01 | 0.01 | 0.01 | 0.00 | 0.00 | 0.00 | 0.01 |
| Proteobacteria | Phyllobacteriaceae | *genus_NA* | 0.03 | 0.00 | 0.00 | 0.01 | 0.00 | 0.01 | 0.01 | 0.00 | 0.00 |
| Proteobacteria | Rhizobiaceae | *Kaistia* | 0.16 | 0.00 | 0.00 | 0.00 | 0.00 | 0.00 | 0.00 | 0.00 | 0.00 |
| Proteobacteria | Rhizobiaceae | *Rhizobium* | 0.28 | 0.01 | 0.01 | 0.04 | 0.01 | 0.01 | 0.11 | 0.01 | 0.01 |
| Proteobacteria | Rhodobiaceae | *Tepidamorphus* | 0.01 | 0.01 | 0.00 | 0.00 | 0.00 | 0.01 | 0.01 | 0.01 | 0.01 |
| Proteobacteria | Xanthobacteraceae | *Ancylobacter* | 0.00 | 0.00 | 0.01 | 0.00 | 0.00 | 0.00 | 0.00 | 0.00 | 0.00 |
| Proteobacteria | Xanthobacteraceae | *genus_NA* | 0.08 | 0.04 | 0.02 | 0.02 | 0.02 | 0.02 | 0.04 | 0.02 | 0.00 |
| Proteobacteria | empty_family | *Nordella* | 0.08 | 0.04 | 0.07 | 0.05 | 0.03 | 0.07 | 0.04 | 0.05 | 0.10 |
| Proteobacteria | family_NA | *genus_NA* | 0.26 | 0.18 | 0.11 | 0.11 | 0.15 | 0.23 | 0.23 | 0.37 | 0.22 |
| Proteobacteria | Rhodobacteraceae | *Rhodobacter* | 0.09 | 0.00 | 0.00 | 0.00 | 0.01 | 0.01 | 0.01 | 0.01 | 0.00 |
| Proteobacteria | Rhodobacteraceae | *Rhodovulum* | 0.00 | 0.00 | 0.00 | 0.00 | 0.00 | 0.00 | 0.00 | 0.01 | 0.00 |
| Proteobacteria | Rhodobacteraceae | *Rubellimicrobium* | 0.00 | 0.00 | 0.00 | 0.00 | 0.00 | 0.00 | 0.00 | 0.02 | 0.00 |
| Proteobacteria | Rhodobacteraceae | *Sediminimonas* | 0.00 | 0.00 | 0.00 | 0.00 | 0.00 | 0.00 | 0.00 | 0.01 | 0.00 |
| Proteobacteria | Rhodobacteraceae | *genus_NA* | 0.02 | 0.00 | 0.00 | 0.00 | 0.00 | 0.00 | 0.01 | 0.01 | 0.01 |
| Proteobacteria | Acetobacteraceae | *Acidisphaera* | 0.00 | 0.00 | 0.00 | 0.00 | 0.01 | 0.00 | 0.00 | 0.00 | 0.00 |
| Proteobacteria | Acetobacteraceae | *Roseococcus* | 0.00 | 0.00 | 0.02 | 0.02 | 0.00 | 0.00 | 0.01 | 0.03 | 0.00 |
| Proteobacteria | Acetobacteraceae | *Roseomonas* | 0.03 | 0.00 | 0.00 | 0.01 | 0.00 | 0.00 | 0.03 | 0.00 | 0.00 |
| Proteobacteria | Acetobacteraceae | *genus_NA* | 0.00 | 0.00 | 0.00 | 0.00 | 0.00 | 0.00 | 0.00 | 0.00 | 0.02 |
| Proteobacteria | Rhodospirillaceae | *Defluviicoccus* | 0.38 | 0.42 | 0.38 | 0.38 | 0.43 | 0.62 | 0.45 | 0.43 | 0.64 |
| Proteobacteria | Rhodospirillaceae | *Inquilinus* | 0.00 | 0.00 | 0.00 | 0.01 | 0.00 | 0.00 | 0.00 | 0.00 | 0.00 |
| Proteobacteria | Rhodospirillaceae | *Thalassospira* | 0.00 | 0.01 | 0.00 | 0.00 | 0.00 | 0.00 | 0.00 | 0.00 | 0.00 |
| Proteobacteria | Rhodospirillaceae | *genus_NA* | 0.00 | 0.02 | 0.01 | 0.01 | 0.01 | 0.04 | 0.01 | 0.00 | 0.02 |
| Proteobacteria | empty_family | *Reyranella* | 0.00 | 0.01 | 0.01 | 0.03 | 0.08 | 0.18 | 0.13 | 0.17 | 0.35 |
| Proteobacteria | family_NA | *genus_NA* | 0.00 | 0.00 | 0.00 | 0.00 | 0.00 | 0.00 | 0.00 | 0.00 | 0.02 |
| Proteobacteria | Anaplasmataceae | *genus_NA* | 0.00 | 0.00 | 0.00 | 0.00 | 0.00 | 0.00 | 0.00 | 0.00 | 0.01 |
| Proteobacteria | Rickettsiaceae | *Rickettsia* | 0.02 | 0.00 | 0.00 | 0.00 | 0.00 | 0.00 | 0.00 | 0.00 | 0.00 |
| Proteobacteria | family_NA | *genus_NA* | 0.01 | 0.00 | 0.01 | 0.00 | 0.00 | 0.00 | 0.00 | 0.00 | 0.00 |
| Proteobacteria | Erythrobacteraceae | *Altererythrobacter* | 0.03 | 0.04 | 0.03 | 0.03 | 0.03 | 0.02 | 0.01 | 0.02 | 0.01 |
| Proteobacteria | Erythrobacteraceae | *Erythrobacter* | 0.00 | 0.02 | 0.01 | 0.01 | 0.01 | 0.00 | 0.01 | 0.00 | 0.00 |
| Proteobacteria | Erythrobacteraceae | *Porphyrobacter* | 0.03 | 0.02 | 0.03 | 0.04 | 0.02 | 0.03 | 0.03 | 0.02 | 0.01 |
| Proteobacteria | Erythrobacteraceae | *genus_NA* | 0.01 | 0.00 | 0.00 | 0.00 | 0.00 | 0.01 | 0.00 | 0.00 | 0.00 |
| Proteobacteria | Sphingomonadaceae | *Blastomonas* | 13.99 | 15.11 | 14.79 | 15.58 | 14.86 | 8.96 | 4.51 | 1.81 | 2.30 |
| Proteobacteria | Sphingomonadaceae | *Novosphingobium* | 0.52 | 2.51 | 1.72 | 2.15 | 2.06 | 3.50 | 3.42 | 1.82 | 2.99 |
| Proteobacteria | Sphingomonadaceae | *Parasphingopyxis* | 0.00 | 0.01 | 0.00 | 0.00 | 0.00 | 0.00 | 0.00 | 0.00 | 0.00 |
| Proteobacteria | Sphingomonadaceae | *Sandarakinorhabdus* | 0.00 | 0.01 | 0.01 | 0.01 | 0.01 | 0.00 | 0.01 | 0.03 | 0.02 |
| Proteobacteria | Sphingomonadaceae | *Sphingobium* | 0.05 | 0.04 | 0.05 | 0.05 | 0.04 | 0.03 | 0.02 | 0.03 | 0.01 |
| Proteobacteria | Sphingomonadaceae | *Sphingomonas* | 0.11 | 0.06 | 0.07 | 0.06 | 0.02 | 0.05 | 0.05 | 0.04 | 0.01 |
| Proteobacteria | Sphingomonadaceae | *Sphingopyxis* | 0.21 | 0.08 | 0.05 | 0.07 | 0.03 | 0.03 | 0.06 | 0.03 | 0.05 |
| Proteobacteria | Sphingomonadaceae | *Sphingorhabdus* | 0.01 | 0.01 | 0.00 | 0.01 | 0.01 | 0.02 | 0.00 | 0.00 | 0.00 |
| Proteobacteria | Sphingomonadaceae | *Zymomonas* | 0.00 | 0.01 | 0.02 | 0.00 | 0.00 | 0.01 | 0.00 | 0.00 | 0.01 |
| Proteobacteria | Sphingomonadaceae | *genus_NA* | 0.19 | 0.17 | 0.13 | 0.18 | 0.13 | 0.14 | 0.14 | 0.09 | 0.02 |
| Proteobacteria | family_NA | *genus_NA* | 0.25 | 0.28 | 0.29 | 0.20 | 0.14 | 0.20 | 0.13 | 0.07 | 0.02 |
| Proteobacteria | empty_family | *Geminicoccus* | 0.06 | 0.00 | 0.00 | 0.00 | 0.00 | 0.00 | 0.01 | 0.00 | 0.00 |
| Proteobacteria | family_NA | *genus_NA* | 0.01 | 0.01 | 0.01 | 0.00 | 0.00 | 0.01 | 0.01 | 0.01 | 0.00 |
| Proteobacteria | Alcaligenaceae | *genus_NA* | 0.00 | 0.00 | 0.00 | 0.00 | 0.01 | 0.00 | 0.01 | 0.00 | 0.00 |
| Proteobacteria | Burkholderiaceae | *Burkholderia* | 0.20 | 0.08 | 0.09 | 0.06 | 0.13 | 0.18 | 1.24 | 1.40 | 1.33 |
| Proteobacteria | Burkholderiaceae | *Pandoraea* | 0.00 | 0.00 | 0.01 | 0.00 | 0.00 | 0.00 | 0.02 | 0.01 | 0.01 |
| Proteobacteria | Burkholderiaceae | *Ralstonia* | 2.77 | 3.41 | 2.99 | 3.25 | 2.58 | 3.02 | 3.27 | 1.63 | 2.00 |
| Proteobacteria | Burkholderiaceae | *genus_NA* | 0.00 | 0.00 | 0.00 | 0.00 | 0.00 | 0.00 | 0.01 | 0.01 | 0.00 |
| Proteobacteria | Comamonadaceae | *Acidovorax* | 0.02 | 0.01 | 0.00 | 0.02 | 0.00 | 0.01 | 0.03 | 0.02 | 0.01 |
| Proteobacteria | Comamonadaceae | *Albidiferax* | 0.11 | 0.07 | 0.07 | 0.07 | 0.04 | 0.04 | 0.05 | 0.06 | 0.02 |
| Proteobacteria | Comamonadaceae | *Aquabacterium* | 0.17 | 0.07 | 0.09 | 0.06 | 0.04 | 0.05 | 0.05 | 0.07 | 0.00 |
| Proteobacteria | Comamonadaceae | *Brachymonas* | 0.00 | 0.00 | 0.00 | 0.00 | 0.00 | 0.00 | 0.01 | 0.02 | 0.01 |
| Proteobacteria | Comamonadaceae | *Caenimonas* | 0.04 | 0.01 | 0.03 | 0.02 | 0.02 | 0.01 | 0.00 | 0.01 | 0.01 |
| Proteobacteria | Comamonadaceae | *Caldimonas* | 0.01 | 0.01 | 0.00 | 0.01 | 0.00 | 0.01 | 0.01 | 0.02 | 0.00 |
| Proteobacteria | Comamonadaceae | *Comamonas* | 0.01 | 0.00 | 0.00 | 0.00 | 0.00 | 0.00 | 0.00 | 0.00 | 0.00 |
| Proteobacteria | Comamonadaceae | *Delftia* | 12.79 | 4.60 | 4.07 | 4.02 | 3.04 | 3.60 | 1.98 | 1.42 | 1.48 |
| Proteobacteria | Comamonadaceae | *Diaphorobacter* | 0.00 | 0.01 | 0.00 | 0.00 | 0.00 | 0.00 | 0.00 | 0.00 | 0.00 |
| Proteobacteria | Comamonadaceae | *Hydrogenophaga* | 0.01 | 0.00 | 0.01 | 0.02 | 0.02 | 0.01 | 0.03 | 0.05 | 0.01 |
| Proteobacteria | Comamonadaceae | *Leptothrix* | 0.15 | 0.14 | 0.07 | 0.08 | 0.04 | 0.05 | 0.08 | 0.06 | 0.02 |
| Proteobacteria | Comamonadaceae | *Limnohabitans* | 0.03 | 0.01 | 0.00 | 0.01 | 0.00 | 0.00 | 0.00 | 0.00 | 0.00 |
| Proteobacteria | Comamonadaceae | *Paucibacter* | 0.02 | 0.01 | 0.00 | 0.00 | 0.00 | 0.00 | 0.00 | 0.01 | 0.00 |
| Proteobacteria | Comamonadaceae | *Pelomonas* | 0.10 | 0.03 | 0.01 | 0.01 | 0.00 | 0.03 | 0.01 | 0.02 | 0.01 |
| Proteobacteria | Comamonadaceae | *Pseudorhodoferax* | 0.02 | 0.01 | 0.00 | 0.01 | 0.02 | 0.01 | 0.04 | 0.04 | 0.15 |
| Proteobacteria | Comamonadaceae | *Rhizobacter* | 0.20 | 0.10 | 0.11 | 0.11 | 0.06 | 0.06 | 0.06 | 0.05 | 0.01 |
| Proteobacteria | Comamonadaceae | *Rivibacter* | 0.00 | 0.01 | 0.00 | 0.00 | 0.00 | 0.00 | 0.00 | 0.00 | 0.00 |
| Proteobacteria | Comamonadaceae | *Roseateles* | 0.08 | 0.08 | 0.07 | 0.06 | 0.06 | 0.06 | 0.06 | 0.06 | 0.02 |
| Proteobacteria | Comamonadaceae | *Variovorax* | 0.03 | 0.03 | 0.00 | 0.01 | 0.01 | 0.01 | 0.00 | 0.03 | 0.01 |
| Proteobacteria | Comamonadaceae | *genus_NA* | 0.41 | 0.26 | 0.25 | 0.26 | 0.22 | 0.24 | 0.22 | 0.21 | 0.10 |
| Proteobacteria | Oxalobacteraceae | *Collimonas* | 0.06 | 0.08 | 0.16 | 0.09 | 0.09 | 0.10 | 0.09 | 0.10 | 0.04 |
| Proteobacteria | Oxalobacteraceae | *Herbaspirillum* | 0.00 | 0.00 | 0.00 | 0.00 | 0.00 | 0.01 | 0.01 | 0.00 | 0.00 |
| Proteobacteria | Oxalobacteraceae | *Massilia* | 0.10 | 0.12 | 0.06 | 0.05 | 0.07 | 0.08 | 0.07 | 0.07 | 0.01 |
| Proteobacteria | Oxalobacteraceae | *Noviherbaspirillum* | 0.62 | 0.36 | 0.42 | 0.43 | 0.33 | 0.36 | 0.49 | 0.54 | 0.23 |
| Proteobacteria | Oxalobacteraceae | *Oxalicibacterium* | 0.12 | 0.05 | 0.09 | 0.08 | 0.04 | 0.08 | 0.08 | 0.04 | 0.02 |
| Proteobacteria | Oxalobacteraceae | *Paucimonas* | 0.00 | 0.00 | 0.00 | 0.00 | 0.00 | 0.00 | 0.01 | 0.01 | 0.00 |
| Proteobacteria | Oxalobacteraceae | *Undibacterium* | 0.00 | 0.01 | 0.01 | 0.00 | 0.00 | 0.00 | 0.00 | 0.00 | 0.00 |
| Proteobacteria | Oxalobacteraceae | *genus_NA* | 0.20 | 0.15 | 0.15 | 0.14 | 0.08 | 0.16 | 0.12 | 0.11 | 0.03 |
| Proteobacteria | family_NA | *genus_NA* | 0.02 | 0.02 | 0.03 | 0.00 | 0.02 | 0.02 | 0.03 | 0.02 | 0.00 |
| Proteobacteria | Ferritrophicaceae | *Ferritrophicum* | 0.01 | 0.00 | 0.00 | 0.00 | 0.00 | 0.00 | 0.00 | 0.00 | 0.00 |
| Proteobacteria | Methylophilaceae | *Methylophilus* | 0.00 | 0.00 | 0.00 | 0.00 | 0.00 | 0.00 | 0.01 | 0.00 | 0.00 |
| Proteobacteria | Gallionellaceae | *Sideroxydans* | 0.00 | 0.00 | 0.02 | 0.00 | 0.00 | 0.00 | 0.00 | 0.00 | 0.00 |
| Proteobacteria | Gallionellaceae | *genus_NA* | 0.03 | 0.01 | 0.00 | 0.01 | 0.00 | 0.00 | 0.01 | 0.01 | 0.00 |
| Proteobacteria | Nitrosomonadaceae | *genus_NA* | 0.01 | 0.01 | 0.00 | 0.00 | 0.01 | 0.00 | 0.00 | 0.00 | 0.00 |
| Proteobacteria | Rhodocyclaceae | *Azoarcus* | 0.01 | 0.03 | 0.02 | 0.01 | 0.01 | 0.01 | 0.00 | 0.01 | 0.01 |
| Proteobacteria | Rhodocyclaceae | *Azospira* | 0.00 | 0.01 | 0.02 | 0.01 | 0.00 | 0.00 | 0.00 | 0.01 | 0.00 |
| Proteobacteria | Rhodocyclaceae | *Dechloromonas* | 0.03 | 0.05 | 0.03 | 0.01 | 0.01 | 0.01 | 0.00 | 0.00 | 0.00 |
| Proteobacteria | Rhodocyclaceae | *Methyloversatilis* | 2.53 | 1.87 | 2.63 | 1.94 | 3.20 | 2.18 | 1.12 | 1.29 | 1.77 |
| Proteobacteria | Rhodocyclaceae | *Propionivibrio* | 0.00 | 0.01 | 0.00 | 0.00 | 0.00 | 0.00 | 0.00 | 0.01 | 0.00 |
| Proteobacteria | Rhodocyclaceae | *Uliginosibacterium* | 0.00 | 0.00 | 0.00 | 0.01 | 0.00 | 0.00 | 0.00 | 0.00 | 0.00 |
| Proteobacteria | Rhodocyclaceae | *Zoogloea* | 0.00 | 0.00 | 0.01 | 0.01 | 0.00 | 0.00 | 0.00 | 0.00 | 0.00 |
| Proteobacteria | Rhodocyclaceae | *genus_NA* | 0.12 | 0.09 | 0.09 | 0.06 | 0.07 | 0.07 | 0.06 | 0.05 | 0.02 |
| Proteobacteria | family_NA | *genus_NA* | 0.08 | 0.01 | 0.04 | 0.05 | 0.02 | 0.02 | 0.02 | 0.05 | 0.13 |
| Proteobacteria | Bacteriovoracaceae | *Peredibacter* | 0.00 | 0.01 | 0.00 | 0.00 | 0.00 | 0.00 | 0.00 | 0.00 | 0.00 |
| Proteobacteria | Bdellovibrionaceae | *Bdellovibrio* | 0.05 | 0.04 | 0.00 | 0.00 | 0.00 | 0.00 | 0.00 | 0.00 | 0.00 |
| Proteobacteria | Pseudoalteromonadaceae | *Pseudoalteromonas* | 0.00 | 0.00 | 0.00 | 0.00 | 0.01 | 0.01 | 0.00 | 0.00 | 0.00 |
| Proteobacteria | Shewanellaceae | *Shewanella* | 0.00 | 0.00 | 0.00 | 0.01 | 0.01 | 0.01 | 0.01 | 0.00 | 0.01 |
| Proteobacteria | Chromatiaceae | *Nitrosococcus* | 0.04 | 0.02 | 0.01 | 0.01 | 0.01 | 0.00 | 0.01 | 0.01 | 0.01 |
| Proteobacteria | Ectothiorhodospiraceae | *Acidiferrobacter* | 0.02 | 0.01 | 0.00 | 0.00 | 0.00 | 0.00 | 0.00 | 0.00 | 0.01 |
| Proteobacteria | Thioalkalispiraceae | *Thiohalophilus* | 0.02 | 0.00 | 0.00 | 0.00 | 0.00 | 0.00 | 0.00 | 0.01 | 0.00 |
| Proteobacteria | Moraxellaceae | *Perlucidibaca* | 1.88 | 0.53 | 0.22 | 0.31 | 0.30 | 0.31 | 0.23 | 0.20 | 0.42 |
| Proteobacteria | Moraxellaceae | *Psychrobacter* | 0.00 | 0.00 | 0.00 | 0.00 | 0.00 | 0.00 | 0.00 | 0.06 | 0.00 |
| Proteobacteria | Pseudomonadaceae | *Pseudomonas* | 0.30 | 0.01 | 0.04 | 0.01 | 0.01 | 0.00 | 0.01 | 0.00 | 0.00 |
| Proteobacteria | Vibrionaceae | *Aliivibrio* | 0.00 | 0.00 | 0.00 | 0.00 | 0.00 | 0.00 | 0.00 | 0.00 | 0.01 |
| Proteobacteria | Vibrionaceae | *Photobacterium* | 0.03 | 0.01 | 0.00 | 0.02 | 0.00 | 0.01 | 0.00 | 0.00 | 0.01 |
| Verrucomicrobia | Verrucomicrobiaceae | *Prosthecobacter* | 0.98 | 0.68 | 0.30 | 0.32 | 0.32 | 0.41 | 0.23 | 0.34 | 1.49 |
| phylum_NA | family_NA | *genus_NA* | 0.10 | 0.28 | 0.23 | 0.33 | 0.60 | 0.86 | 0.24 | 0.09 | 0.47 |
